# Haemodynamic changes and mean airway pressure threshold in extremely preterm infants (22–24 weeks of gestation) with tension pneumothorax

**DOI:** 10.1016/j.resplu.2025.100954

**Published:** 2025-04-08

**Authors:** Tomonori Kurimoto, Takuya Tokuhisa, Asataro Yara, Masaya Kibe, Hiroshi Ohashi, Masakatsu Yamamoto, Tsuyoshi Yamamoto, Eiji Hirakawa

**Affiliations:** Department of Neonatology, Perinatal Medical Center, Kagoshima City Hospital, 37-1 Uearata, Kagoshima 890-8760, Japan

**Keywords:** Tension pneumothorax, Extremely preterm infants, Central venous pressure, Mean airway pressure

## Abstract

**Background:**

Extremely preterm infants (22–24 weeks of gestation) are at high risk of tension pneumothorax, a life-threatening condition that causes haemodynamic instability. This study aimed to analyse the haemodynamic changes associated with tension pneumothorax and identify the mean airway pressure (MAP) threshold associated with its onset.

**Methods:**

This retrospective descriptive study was conducted in a Level III Neonatal Intensive Care Unit (NICU) (2014–2024). Infants born between 22 + 0 to 24 + 6 weeks of gestation who developed tension pneumothorax within 72 h of birth were included. Haemodynamic parameters, including central venous pressure (CVP), mean arterial pressure (mBP), heart rate (HR), saturation of percutaneous oxygen (SpO_2_), fraction of inspired oxygen (FiO_2_), and MAP, were analysed at baseline, pneumothorax onset, and post-decompression. Statistical analyses included the Wilcoxon signed-rank test and generalised linear mixed model (GLMM).

**Results:**

The CVP significantly increased at the onset of pneumothorax (*p* = 0.004) and decreased post-intervention (*p* = 0.004). The mBP and HR declined at onset (*p* = 0.0005) and partially recovered post-intervention. MAP was significantly higher at onset (*p* = 0.003), and GLMM analysis showed that an MAP increase of ≥1.3 cmH_2_O was associated with pneumothorax risk.

**Conclusions:**

A sudden increase in CVP may serve as an early indicator of tension pneumothorax. An MAP threshold of ≥12 cmH_2_O was linked to higher pneumothorax risk, emphasising the need for cautious ventilatory management. Persistent haemodynamic instability after decompression highlights the need for close monitoring. Further studies are required to refine the neonatal care strategies.

## Highlights

• Sudden CVP increase may be an early indicator of tension pneumothorax.
• An MAP threshold ≥12 cmH_2_O is linked to higher pneumothorax risk.
• Hemodynamic instability often persists even after decompression therapy.
• Continuous CVP monitoring may help in the early detection of pneumothorax.
• Further studies needed to refine neonatal ventilatory management strategies.

## Introduction

Extremely preterm infants (22–24 weeks of gestation) are at high risk of developing tension pneumothorax, a life-threatening complication characterised by elevated intrathoracic pressure, impaired venous return, and haemodynamic instability. The incidence of pneumothorax within the first 72 h of life is reported to be 9.2% in infants born at 23–28 weeks of gestation.^1^ However, at our institution, the incidence is higher, at 14.1% among infants born at 22–23 weeks, with 71.4% of cases occurring within this period. This condition significantly increased mortality risk (odds ratio [OR]: 5.88, 95% confidence interval [CI]: 1.57–22.0).^2^ While antenatal corticosteroids (risk ratio [RR]: 0.76, 95% CI: 0.32–1.80) and surfactant therapy may reduce the risk, infants born at 22–24 weeks remain highly vulnerable, highlighting the need for more effective preventive strategies. Despite advances in neonatal care, the mechanisms and risk factors that contribute to tension pneumothorax, particularly the mean airway pressure (MAP) threshold associated with its onset, remain unclear.^3^, ^4^

Central venous pressure (CVP) monitoring using an umbilical venous catheter (UVC) is an essential tool for assessing right atrial pressure and haemodynamic status.^5^ Although CVP elevation is associated with increased intrathoracic pressure, its role in pneumothorax has not been systematically investigated, and limited data are available on the haemodynamic changes before, during, and after its onset in extremely preterm infants.

To address these gaps, this study examined haemodynamic changes associated with tension pneumothorax in extremely preterm infants. We analysed the CVP, mean arterial blood pressure (mBP), heart rate (HR), saturation of percutaneous oxygen (SpO_2_), fraction of inspired oxygen (FiO_2_), and MAP at three time points: baseline (prior to pneumothorax onset), pre-decompression (at diagnosis), and post-decompression (following intervention). By comparing these time points, we aimed to delineate the progression of haemodynamic instability and extent of recovery after decompression. We also determined the MAP threshold associated with the development of pneumothorax. The findings of this study will enhance our understanding of its haemodynamic impact and help inform strategies for early detection and intervention. Given the rarity of the condition and the limited sample size, the present investigation was conducted as a retrospective descriptive study aimed at characterising these haemodynamic changes and identifying potential clinical indicators of tension pneumothorax.

## Methods

### Study design and population

This retrospective descriptive study was conducted in the Level III neonatal intensive care unit (NICU) of Kagoshima City Hospital between January 2014 and December 2024. It included extremely preterm infants born between 22 weeks + 0 days and 24 weeks + 6 days of gestation who developed tension pneumothorax within 72 h of birth. This study was conducted and reported in accordance with the STROBE (Strengthening the Reporting of Observational Studies in Epidemiology) guidelines.

### Inclusion and exclusion criteria

Infants who had respiratory distress syndrome (RDS) requiring surfactant therapy, a functioning UVC with continuous CVP monitoring, and a confirmed diagnosis of tension pneumothorax based on clinical signs (tachycardia, bradycardia, hypotension, and desaturation) and radiographic findings (lung displacement, diaphragmatic depression, contralateral mediastinal shift, or transillumination) were included. Only infants with documented CVP values before, during, and after intervention were included. Infants who had malpositioned or dysfunctional UVC or major congenital anomalies were excluded.

### Clinical management of tension pneumothorax

Tension pneumothorax was managed according to a standardised institutional protocol, which included immediate needle thoracentesis for decompression, followed by chest tube placement when necessary. In all 12 cases included in this study, initial decompression was performed by needle thoracentesis; however, none of the cases resolved with needle aspiration alone, and all required subsequent chest tube placement.

### Data collection

Clinical data were retrospectively retrieved from electronic medical records using FileMaker Pro (Claris International Inc., Santa Clara, CA, USA). Haemodynamic and respiratory parameters were continuously monitored using NICU bedside monitors (CSM-1901; Life Scope G; Nihon Kohden, Tokyo, Japan) with high-fidelity data recording.

The following parameters were analysed: CVP, mBP, HR, SpO_2_, FiO_2_, and MAP. These parameters were assessed at three time points: before the onset of pneumothorax (1–3 h prior), at the time of diagnosis (immediately before thoracic decompression), and after successful intervention (1–3 h post-decompression). In our NICU, CVP monitoring is routinely performed in infants born at 22–24 weeks of gestation using UVCs as the initial central venous access. This practice is part of our standard haemodynamic monitoring protocol and enabled reliable collection of CVP data in the present study.

### Umbilical venous catheter (UVC) placement

The UVC was introduced through the umbilical vein, advanced through the left portal vein, ductus venosus, and hepatic vein, and terminated in the inferior vena cava (IVC) or right atrium. The ideal catheter tip position was confirmed by radiography, typically at the IVC-right atrial junction between T7 and T9.^6^

### Ventilatory management

Upon admission, the infants were initially ventilated using the synchronised intermittent mandatory ventilation (SIMV) mode (VN500 ventilator; Dräger, Germany), with FiO_2_ adjusted to maintain postductal SpO_2_ at 90–95%.^7^, ^8^ All patients received surfactant therapy within the first hour of life for RDS. Volume guarantee (VG) mode was not employed during SIMV. If respiratory acidosis (pH <7.2, PaCO_2_ >65 mmHg) persisted despite SIMV settings (peak inspiratory pressure (PIP): 20–21 cmH_2_O, positive end-expiratory pressure (PEEP): 5–6 cmH_2_O, respiratory rate (RR): 60–65 breaths/min), infants were switched to high-frequency oscillatory ventilation (HFOV) when PaCO_2_ failed to decrease by >10% and/or FiO_2_ by >20% within 1 h.^9^, ^10^ HFOV was initiated with MAP set 2–3 cmH_2_O above that used during SIMV and was subsequently adjusted as needed. The oscillation frequency was set to 12–15 Hz and was reduced to 10 Hz if necessary. The I:E ratio was maintained at 1:1. During HFOV, VG mode was applied with a target range of 1.5–2.0 mL/kg.^11^ There was no apparent difference in pneumothorax incidence between infants initially managed with SIMV and those who were switched to HFOV.

### Statistical analysis

Given the small sample size, descriptive statistics were the primary analytical approach. Comparisons across the three time points (pre-onset, onset, and post-intervention) were performed using the Wilcoxon signed-rank test for paired non-parametric data. The Hodges–Lehmann method was used to estimate median differences with 95% confidence intervals (CIs), and rank-biserial correlation was applied to assess the magnitude of effect sizes. To further explore time-dependent changes and account for individual variability, a generalised linear mixed model (GLMM) was constructed as a supplementary analysis. This model included measurement time and gestational age as fixed effects, and subject ID as a random intercept. The model was estimated using restricted maximum likelihood (REML) to ensure unbiased variance estimation. Although statistical power was limited due to the small sample, the GLMM results were consistent with descriptive findings and may offer a reproducible framework for hypothesis generation in future studies.

Statistical significance was defined as *p* < 0.05. All analyses were conducted using R (R Foundation for Statistical Computing, Vienna, Austria) and JMP 14 (SAS Institute, Inc., Cary, NC, USA).

## Results

Of the 12 patients included in the study, the median onset time of symptoms was 27 h after birth (interquartile range [IQR], 11–48 h) (Table 1). All infants were similarly critically ill at baseline, with comparable Apgar scores and severity of respiratory distress syndrome. During the study period (2014–2024), a total of 192 infants were born at 22–24 weeks of gestation in our NICU. Among these, 19 developed tension pneumothorax within 72 h of birth, and CVP monitoring was attempted in all cases via an UVC. However, CVP data were complete for only 12 infants, who were therefore included in the final analysis. These cases were distributed evenly throughout the study period, indicating no temporal clustering or bias related to year of occurrence. Haemodynamic changes associated with tension pneumothorax were evaluated using the Wilcoxon signed-rank test and Hodges–Lehmann 95% confidence intervals. Comparisons of CVP, mBP, HR, SpO_2_, FiO_2_, and MAP across the three time points (pre-onset [baseline], onset of tension pneumothorax, and post-intervention) revealed significant changes. These alterations were particularly pronounced between the pre- and post-intervention onset of tension pneumothorax as well as between the onset of tension pneumothorax and post-intervention, indicating substantial haemodynamic disturbances during tension pneumothorax and subsequent recovery following intervention.

**Table 1:** Clinical characteristics of infants with tension pneumothorax.

	N, ％ or Interquartile Range
Sex (male)	6 (50)
Birth weight (median)	602 (466–662)
Weeks(median)	23 (23–24)
22w	3 (25%)
FGR (less than or 10%tile)	1 (8.3%)
IVH3-4 72 h	4 (33.3%)
EOS	1 (8.3%)
mortality	5 (41.7%)
Out-of-hospital birth	1 (8.3%)
C/S	11(91.7%)
PROM	2 (16.7%)
CAM stage 2–3	5 (41.7%)
funisitis stage 2–3	3 (25%)
Oligohydramnios	2 (16.7%)
Antenatal steroid	3 (25%)
APS 1 min/5min	2 (1–3)/ 6 (3–6)
UA pH	7.32 (7.17–7.36)
RDS	12 (100%)
Fetal bradycardia	1 (8.3%)
Fentanyl	3 (25%)
Vecuronium	1 (8.3%)
DEX	1 (8.3%)
PB	5 (41.7%)

This table presents the demographic data and clinical characteristics of study participants who developed tension pneumothorax within 72 h of birth.

**Abbreviations:** FGR, fetal growth restriction; IVH, intraventricular hemorrhage; EOS, early-onset sepsis; C/S, cesarean section; PROM. premature rupture of membranes; CAM, chorioamnionitis; APS, apgar score; UA, umbilical artery; RDS, respiratory distress syndrome; DEX, dexamethasone; PB, phenobarbital.

### The Wilcoxon signed-rank test and Hodges-Lehmann 95% confidence interval (Table 2)

**Fig. 1:**
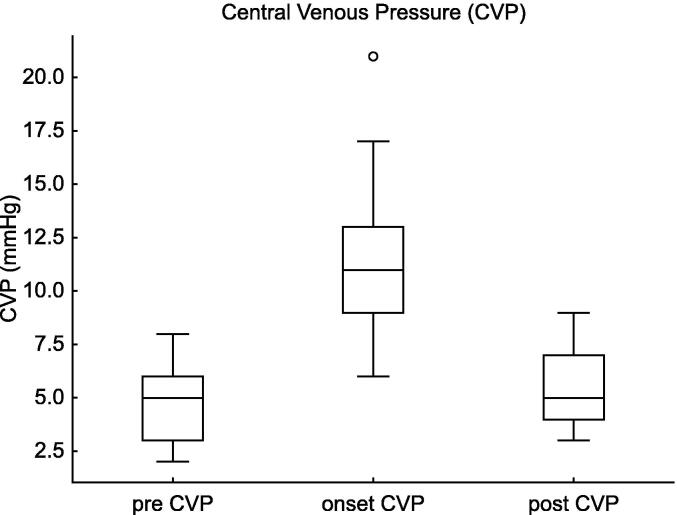
**Central Venous Pressure (CVP) Changes in Tension Pneumothorax**. This figure illustrates the changes in CVP before, at the onset of, and after intervention for tension pneumothorax in extremely preterm infants (22–24 weeks of gestation). The CVP significantly increased at the onset of pneumothorax (Estimate = +6.0, 95% CI: 2.2 to 17.6, p = 0.004) and decreased following decompression therapy (Estimate = -5.0, 95% CI: -13.2 to -2.2, p = 0.004), highlighting the hemodynamic impact of the condition and the effectiveness of treatment.

The CVP significantly increased at the onset of tension pneumothorax compared to baseline (*p* = 0.004, estimate = +6.0, 95% CI: 2.2–17.6). Following the intervention, CVP decreased significantly (*p* = 0.004, estimate = −5.0, 95% CI: −13.2–−2.2). (Fig. 1).

**Table 2:** Haemodynamic and respiratory changes in infants with tension pneumothorax.

Pre vs. Onset	estimate	95%CI	p-value
CVP	6	2.2–17.6	0.004
m BP	−6.0	−19.5–−2.0	0.0005
HR	−55.0	−124.8–−5.8	0.0005
SpO_2_	−10.5	−53.7–−1.3	0.0005
FiO_2_	0.31	0.10–0.77	0.005
MAP	1.0	0.5–3.0	0.003

Onset vs. Post	estimate	95%CI	p-value
CVP	−5.0	−13.2–−2.2	0.004
mBP	5.5	3.3–20.7	0.007
HR	49.5	12.0–127.8	0.005
SpO_2_	12.5	3.6–50.1	0.0005
FiO_2_	−0.16	−0.10–0.50	0.10
MAP	−1.0	−2.5–1.0	0.10

This table presents the results of the Wilcoxon Signed-Rank Test comparing haemodynamic (CVP, mBP, HR, MAP) and respiratory (SpO_2_, FiO_2_) parameters between the pre-onset and onset of tension pneumothorax, as well as between the onset and post-intervention phases. Significant changes in CVP, mBP, HR, and SpO_2_ were observed, demonstrating the physiological impact of tension pneumothorax and the effectiveness of decompression therapy.

**Abbreviations:** CVP, central venous pressure; mBP, mean blood pressure; HR, heart rate; SpO_2_, saturation of percutaneous oxygen; FiO_2_, fraction of inspiratory oxygen; MAP, mean airway pressure.

**Fig. 2:**
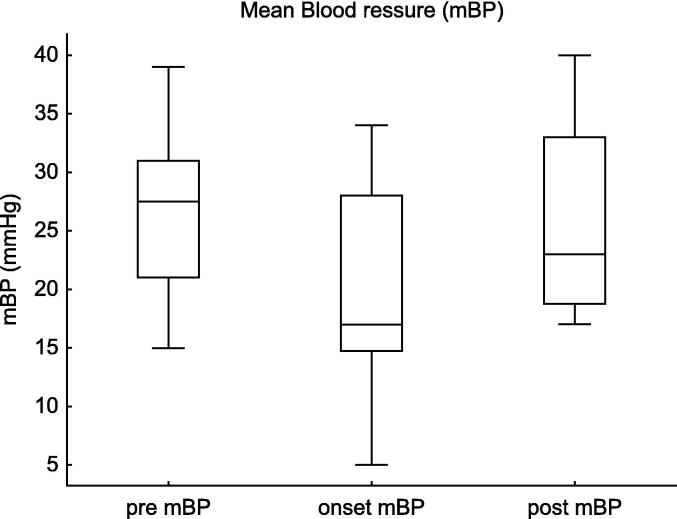
**Mean Blood Pressure (mBP) Changes in Tension Pneumothorax**. This figure shows the changes in mean blood pressure (mBP) before, at the onset of, and after intervention for tension pneumothorax in extremely preterm infants (22–24 weeks of gestation). The mBP significantly decreased at the onset of pneumothorax (Estimate = −6.0, 95% CI: −19.5 to −2.0, p = 0.0005), while post-intervention changes showed a trend toward improvement (Estimate = +5.5, 95% CI: +3.3 to 20.7, p = 0.007).

The mBP showed a marked decline at the onset of pneumothorax compared to baseline (*p* = 0.0005, estimate = −6.0, 95% CI: −19.5–−2.0). Post-intervention, mBP improved significantly (*p* = 0.007, estimate = +5.5, 95% CI: 3.3–20.7). (Fig. 2).

**Fig. 3:**
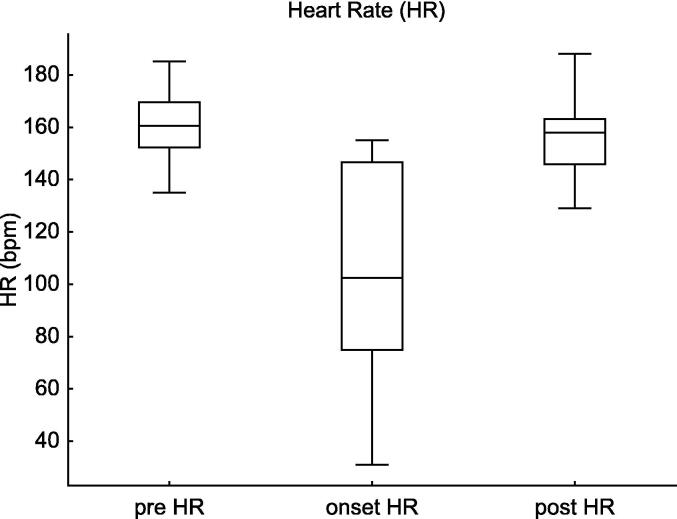
**Heart Rate (HR) Changes in Tension Pneumothorax**. This figure shows the changes in HR before, at the onset of, and after intervention for tension pneumothorax in extremely preterm infants (22–24 weeks of gestation). The HR significantly decreased at the onset of pneumothorax (Estimate = −55.0, 95% CI: −124.8 to −5.8, p = 0.0005) and showed a statistically significant improvement after intervention (Estimate = +49.5, 95% CI: +12.0 to 127.8, p = 0.005).

The HR significantly decreased at the onset of tension pneumothorax compared to baseline (*p* = 0.0005, estimate = −55.0, 95% CI: −124.8–-5.8). Following decompression, HR recovered significantly (*p* = 0.005, estimate = +49.5, 95% CI: 12.0–127.8). (Fig. 3).

**Fig. 4:**
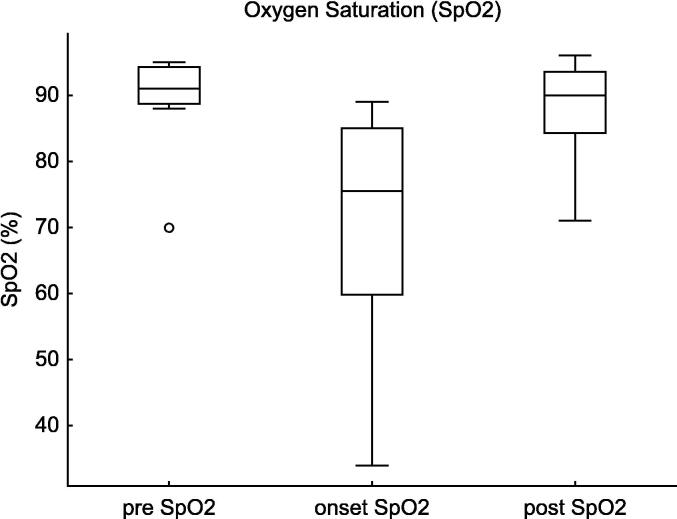
**Oxygen Saturation (SpO_2_) Changes in Tension Pneumothorax**. This figure shows the changes in peripheral SpO_2_ before, at the onset of, and after intervention for tension pneumothorax in extremely preterm infants (22–24 weeks of gestation). SpO_2_ significantly decreased at the onset of pneumothorax (Estimate = −10.5, 95% CI: −53.7 to −1.3, p = 0.0005) and showed a statistically significant improvement following decompression therapy (Estimate = +12.5, 95% CI: 3.6 to 50.1, p = 0.0005).

The SpO_2_ significantly declined at pneumothorax onset (*p* = 0.0005, estimate = −10.5, 95% CI: −53.7–−1.3). Post-intervention, SpO_2_ showed a trend toward improvement (*p* = 0.0005, estimate = +12.5, 95% CI: 3.6–50.1). (Fig. 4).

**Fig. 5:**
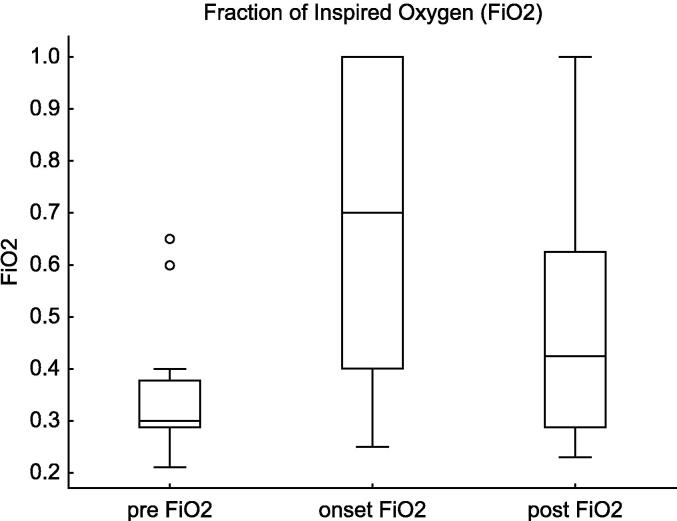
**Fraction of inspired oxygen (FiO_2_) Changes in Tension Pneumothorax**. This figure shows the changes in the FiO₂ before, at the onset of, and after intervention for tension pneumothorax in extremely preterm infants (22–24 weeks of gestation). FiO_2_ significantly increased at the onset of pneumothorax (Estimate = +0.31, 95% CI: 0.10 to 0.77, p = 0.005), but the post-intervention decrease was not statistically significant (95% CI: −0.1 to 0.50, p = 0.10).

The FiO_2_ increased significantly at the onset of pneumothorax (p = 0.005, estimate = +0.31, 95% CI: 0.10–0.77). After the intervention, FiO_2_ decreased (p = 0.1, estimate = -0.16, 95% CI: −0.1–0.50). (Fig. 5).

**Fig. 6:**
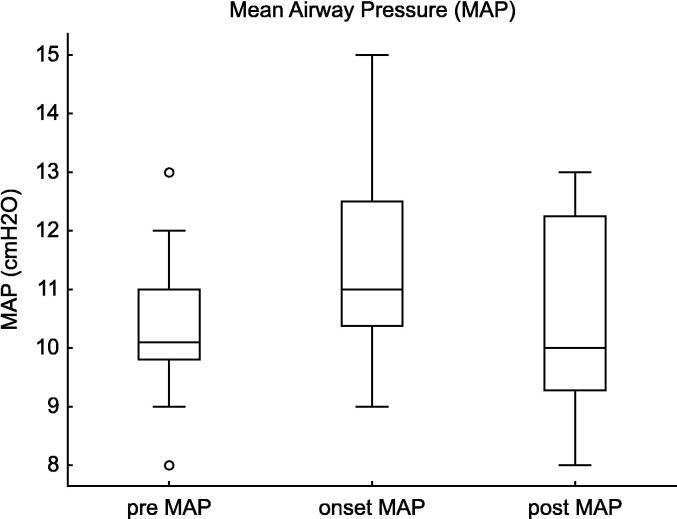
**Mean airway pressure (MAP) Changes in Tension Pneumothorax**. This figure illustrates the changes in MAP before, at the onset of, and after intervention for tension pneumothorax in extremely preterm infants (22–24 weeks of gestation). MAP significantly increased at the onset of pneumothorax (Estimate = +1.0, 95% CI: 0.5 to 3.0, p = 0.003), while the post-intervention decrease did not reach statistical significance (95% CI: −2.5 to 1.0, p = 0.10).

The MAP increased at the onset of pneumothorax compared to baseline (p = 0.003, estimate = +1.0, 95% CI: 0.5–3.0). Post-intervention, MAP showed a trend toward improvement, but the difference was not statistically significant (p = 0.10, estimate = -1.0, 95% CI: −2.5–1.0). (Fig. 6).

### Rank-Biserial correlation (Table 3)

The effect size for pre-vs. onset comparisons was 1.0, indicating a strong association between pneumothorax onset and haemodynamic deterioration. The onset vs. post- comparisons showed partial recovery, with effect sizes of 1.0 for CVP, MAP and FiO2, 0.85 for mBP and SpO_2_, and 0.87 for HR, suggesting substantial but incomplete recovery following intervention.

**Table 3:** Effect sizes of parameter changes in tension pneumothorax.

Pre vs. Onset	Effect Size	Onset vs. Post	Effect Size
CVP	1.00	CVP	1.00
mBP	1.00	mBP	0.85
HR	1.00	HR	0.87
SpO_2_	1.00	SpO_2_	0.85
FiO_2_	1.00	FiO_2_	1.00
MAP	1.00	MAP	1.00

This table shows the Rank-Biserial Correlation effect sizes for haemodynamic (CVP, mBP, HR, MAP) and respiratory (SpO_2_, FiO_2_) parameters. Pre-onset to onset comparisons showed strong deterioration (effect size = 1.0), while onset to post-intervention comparisons indicated partial recovery, with effect sizes ranging from 0.85 to 1.0.

**Abbreviations:** CVP, central venous pressure; mBP, mean blood pressure; HR, heart rate; SpO_2_, saturation of percutaneous oxygen; FiO_2_, fraction of inspiratory oxygen; MAP, mean airway pressure.

### GLMM analysis (Table 4)

The GLMM confirmed that the measurement time was a significant factor influencing CVP, mBP, HR, SpO_2_, FiO_2_, and MAP.

**Table 4:** GLMM analysis of changes in tension pneumothorax.

CVP	Comparison	Estimate	95%CI	p-value
	Intercept	29.0	−8.4–66.5	0.13
	Pre vs. Onset	7.2	4.6–9.8	<0.0001
	Onset vs. Post	−6.2	−11.4–−1.1	0.0008
	Weeks	−1.1	−2.7–0.6	0.20
	Random Variance	0.3	−0.5–1.0	0.49
**mBP**	**Comparison**	**Estimate**	**95%CI**	**p-value**

	Intercept	−55.7	−174.1–62.7	0.36
	Pre vs. Onset	−7.8	−11.3–−4.2	<0.0001
	Onset vs. Post	6.8	−0.4–14.0	0.08
	Weeks	3.6	−1.5–8.7	0.17
	Random Variance	2.2	−0.5–5.0	0.11
**HR**	**Comparison**	**Estimate**	**95%CI**	**p-value**

	Intercept	247.3	−27.1–522.1	0.08
	Pre vs. Onset	−57.5	−80.7–−34.3	<0.0001
	Onset vs. Post	52.4	6.1–98.8	0.002
	Weeks	−3.8	15.6–8.1	0.53
	Random Variance	0.0	−0.4–0.4	1.00
**SpO_2_**	**Comparison**	**Estimate**	**95%CI**	**p-value**

	Intercept	71.1	−84.5–226.7	0.37
	Pre vs. Onset	−20.4	−29.3–−11.6	<0.0001
	Onset vs. Post	18.7	1.1–36.4	0.003
	Weeks	0.8	−5.9–7.5	0.81
	Random Variance	0.4	−0.4–1.2	0.31
**FiO_2_**	**Comparison**	**Estimate**	**95%CI**	**p-value**

	Intercept	0.23	−3.42–3.89	0.90
	Pre vs. Onset	0.33	0.20–0.46	<0.0001
	Onset vs. Post	−0.19	−0.45–0.07	0.10
	Weeks	0.01	−0.15–0.16	0.95
	Random Variance	1.50	−0.44–3.44	0.13
**MAP**	**Comparison**	**Estimate**	**95%CI**	**p-value**

	Intercept	14.1	−12.7–40.8	0.30
	Pre vs. Onset	1.3	0.6–1.9	0.0001
	Onset vs. Post	−1.0	−2.3–0.2	0.10
	Weeks	−0.2	−1.3–1.0	0.78
	Random Variance	3.8	−0.6–8.2	0.09

This table shows the effects of tension pneumothorax and intervention on haemodynamic (CVP, mBP, HR, MAP) and respiratory (SpO_2_, FiO_2_) parameters. Significant changes were observed between pre-onset, onset, and post-intervention phases, highlighting the impact of acute events and treatment response.

**Abbreviations:** CVP, central venous pressure; mBP, mean blood pressure; HR, heart rate; SpO_2_, saturation of percutaneous oxygen; FiO_2_, fraction of inspiratory oxygen; MAP, mean airway pressure.

For CVP, the increase from baseline to pneumothorax onset was statistically significant (estimate = +7.2, 95% CI: 4.6–9.8, *p* < 0.0001), and the subsequent decrease post-intervention was also significant (estimate = −6.2, 95% CI: −11.4–−1.1, *p* = 0.0008).

For mBP, the decline at pneumothorax onset was significant (estimate = −7.8, 95% CI: −11.3–−4.2, *p* < 0.0001), whereas the increase post-intervention was not statistically significant (estimate = +6.8, 95% CI: −0.4–14.0, *p* = 0.08).

For HR, the decrease at onset was significant (estimate = −57.5, 95% CI: −80.7–−34.3, *p* < 0.0001), followed by a significant increase post-intervention (estimate = +52.4, 95% CI: 6.1–98.8, *p* = 0.002).

For SpO_2_, the decline at pneumothorax onset was statistically significant (estimate = −20.4, 95% CI: −29.3–11.6, *p* < 0.0001), but the improvement post-intervention reached significance (estimate = +18.7, 95% CI: 1.1–36.4, *p* = 0.003).

For FiO_2_, the increase at onset was highly significant (estimate = +0.33, 95% CI: 0.20–0.46, *p* < 0.0001), and the decrease post-intervention did not remain statistically significant (estimate = −0.19, 95% CI: −0.45–0.07, *p* = 0.10).

For MAP, the increase at onset was significant (estimate = +1.3, 95% CI: 0.6–1.9, *p* = 0.0001), but the post-intervention decrease was not significant (estimate = −1.0, 95% CI: −2.3–0.2, *p* = 0.10).

Gestational age did not significantly influence changes in CVP, mBP, HR, SpO_2_, FiO2, or MAP, suggesting that pneumothorax-related haemodynamic deterioration is primarily driven by acute events rather than gestational age differences.

GLMM analysis confirmed the time-dependent effects of pneumothorax on haemodynamic parameters, emphasising the critical need for early recognition and intervention in extremely preterm infants.

## Discussion

This study provided a detailed analysis of the haemodynamic impact of tension pneumothorax in extremely preterm infants born at 22–24 weeks of gestation. Key physiological parameters including CVP, mBP, HR, SpO_2_, FiO_2_, and MAP were evaluated at three time points: (i) before the onset of pneumothorax, (ii) at the time of diagnosis, and (iii) after the intervention. These findings highlight the significant physiological changes induced under these critical conditions.

### Haemodynamic changes in tension pneumothorax

The haemodynamic changes observed in this study, including a marked increase in CVP and significant decreases in mBP, HR, and SpO_2_, were consistent with the established pathophysiology of tension pneumothorax. The elevated intrathoracic pressure likely impairs venous return, reduces preload, and ultimately leads to systemic hypotension and hypoxaemia. Several mechanisms may explain the bradycardia observed in this study. First, persistent hypotension may result in reduced coronary perfusion, leading to an inadequate blood supply to the sinoatrial node, impairing its function and subsequently causing bradycardia.^12^ Second, the Bezold–Jarisch reflex (BJR) may have contributed to the observed bradycardia. This cardiovascular depressive reflex is triggered by the abnormal activation of left ventricular stretch receptors.^13^ A paradoxical stimulation of these receptors due to decreased left ventricular filling may have activated vagal sensory neurones, transmitting signals to the area postrema and subsequently to the nucleus of the solitary tract. This process enhances parasympathetic activity while suppressing sympathetic output, ultimately inhibiting sinoatrial node function, resulting in bradycardia.^14^ Additionally, sympathetic inhibition-induced peripheral vasodilation may exacerbate hypotension, reinforcing the haemodynamic changes observed in the present study.

Third, the immaturity of the autonomic nervous system in extremely preterm infants may have contributed to the bradycardia. In the human foetus, parasympathetic inhibition of the sinoatrial node is established between 12 and 17 weeks of gestation, followed by the development of sympathetic innervation between 22 and 24 weeks of gestation.^15^ Consequently, in neonates born at 22–24 weeks of gestation, the parasympathetic nervous system remains dominant over the sympathetic nervous system, predisposing them to vagally mediated sinus bradycardia.

### Clinical implications

A particularly noteworthy finding of this study was the sudden elevation in CVP observed at the onset of tension pneumothorax. This suggests that continuous CVP monitoring using UVC could serve as an early indicator of haemodynamic deterioration. Although CVP has traditionally been used as an indicator of right atrial pressure and for preload assessment, its potential utility in diagnosing tension pneumothorax has not been thoroughly investigated.^16^ Given the rapid progression of this condition, a sudden and sustained increase in CVP should prompt immediate clinical reassessment to rule out pneumothorax as a potential cause. It is worth noting that all patients received surfactant within the first hour of life, which may have influenced early respiratory dynamics but did not prevent the occurrence of pneumothorax. Furthermore, this study demonstrated that even after decompression with chest tube placement for tension pneumothorax treatment, the mBP did not fully recover. This finding suggests that haemodynamic instability may persist in critically ill neonates after tension pneumothorax, potentially due to residual changes in intrathoracic pressure, myocardial dysfunction caused by hypoxaemia and acidosis, or prolonged hypotension. Additionally, while the use of a thin chest drain for tension pneumothorax may reduce pain and trauma during insertion, it may pose a higher risk of blockage in cases of haemothorax, tube kinking, or inadequate air leak evacuation in large pneumothorax.^17^, ^18^ The findings of this study provide valuable insights into the haemodynamic consequences of tension pneumothorax, enhancing our understanding of early diagnostic markers and optimizing intervention strategies.

### Role of mean airway pressure (MAP)

In the present study, an increase in MAP was observed before the onset of tension pneumothorax, which remained elevated at the time of onset. This finding highlights the challenges of optimal ventilatory management in extremely low-birth-weight infants and suggests that excessively high MAP may contribute to barotrauma and increase the risk of alveolar rupture. A previous study reported that extremely preterm infants (gestational age: 25.3 [24.3–26.6] weeks), who required HFOV in the acute phase, had an increased risk of pulmonary air leaks within the first 7 days of life when MAP was ≥13 cmH_2_O.^19^ However, no studies have specifically focused on infants born at 22–24 weeks of gestation. In the present study, MAP at the onset of tension pneumothorax was 11.0 (10.4–12.5) cmH_2_O, and GLMM analysis demonstrated that an increase in MAP by 1.3 cmH_2_O compared to pre-onset levels was associated with a higher risk of pneumothorax. These findings suggest that when MAP exceeds 12 cmH_2_O, clinicians should consider reducing MAP whenever feasible. Identifying a safe MAP threshold is crucial for minimising the iatrogenic risk of pneumothorax, while ensuring adequate gas exchange. Further studies are needed to establish evidence-based ventilatory strategies aimed at reducing the risk of pneumothorax in extremely preterm infants. In addition, a significant increase in FiO_2_ was observed to maintain SpO_2_ at the onset of pneumothorax. These findings highlight the importance of strict monitoring of oxygenation and reflect the systemic effects of pneumothorax on oxygen delivery.

### Limitations

This study had several limitations. First, because this was a retrospective study, it was subject to selection and information biases. However, we used a GLMM to adjust for individual variability and time-dependent effects, thereby minimising the potential bias.

Second, the small sample size (*n* = 12) limited the generalisability and statistical power of our findings. The resulting confidence intervals were wide, and the random effect variance was small, which may reduce the precision of the estimates. Nevertheless, the GLMM results consistently aligned with the descriptive analyses. We interpreted these findings with caution and positioned the GLMM strictly as an exploratory and complementary tool, not for confirmatory inference. To ensure robustness despite the limited sample size, we applied the Wilcoxon signed-rank test and rank-biserial correlation.

Third, although we identified haemodynamic changes associated with pneumothorax, we could not establish causality. Confounding factors such as infection, patent ductus arteriosus, and inotropic support may have influenced our results.

Additionally, our results suggest that MAP ≥ 12 cmH_2_O may increase the risk of tension pneumothorax, but further validation is needed.

Future prospective multicentre studies are necessary to confirm our findings and to improve NICU management strategies for extremely preterm infants.

## Conclusion

This study highlights the haemodynamic impact of tension pneumothorax in extremely preterm infants born at 22–24 weeks of gestation. A sudden increase in CVP may serve as an early indicator of pneumothorax onset, emphasising the potential role of continuous CVP monitoring in early detection. Additionally, an MAP threshold of ≥12 cmH_2_O is associated with an increased risk of pneumothorax, highlighting the need for careful ventilatory management. Persistent post-decompression haemodynamic instability suggests that close monitoring and tailored interventions are necessary, even after successful treatment. Further prospective studies are needed to validate these findings and optimise neonatal care strategies.

## Funding sources

No funding was obtained for this study.

## CRediT authorship contribution statement

**Tomonori Kurimoto:** Writing – original draft, Methodology, Investigation, Formal analysis, Data curation, Conceptualization. **Takuya Tokuhisa:** Writing – review & editing, Supervision. **Asataro Yara:** Conceptualization. **Masaya Kibe:** Conceptualization. **Hiroshi Ohashi:** Conceptualization. **Masakatsu Yamamoto:** Conceptualization. **Tsuyoshi Yamamoto:** Conceptualization. **Eiji Hirakawa:** Conceptualization.

## Declaration of competing interest

The authors declare that they have no known competing financial interests or personal relationships that could have appeared to influence the work reported in this paper.
